# Reducing the burden of iron deficiency anemia in Cote D’Ivoire through fortification

**DOI:** 10.1186/s41043-020-0209-x

**Published:** 2020-02-07

**Authors:** Alberto Prieto-Patron, Zsuzsa V. Hutton, Giovanni Fattore, Magalie Sabatier, Patrick Detzel

**Affiliations:** 1 Nestlé Research Center, Vers-chez-les-Blanc, Lausanne, Switzerland; 2 Department of Policy Analysis and Public Management, CERGAS – Centre for Research in Healthcare Management, Bocconi University, Milan, Italy

**Keywords:** Economic evaluation, Micronutrient fortification, Iron deficiency anemia, Cote d’Ivoire, Model, Impact, DALYs

## Abstract

**Background:**

Iron deficiency anemia (IDA) is highly prevalent in the Cote d’Ivoire and has severe health and economic consequences. In this paper, we apply a health economic model to quantify the burden of IDA, and the contribution of nationwide mandatory iron fortification of wheat flour and voluntary iron fortification of condiments to the reduction of this burden.

**Methods:**

The analysis for the population from 6 months to 64 years builds on published reviews and publicly available datasets and is stratified by age-groups and socioeconomic strata using comparative risk assessment model.

**Results:**

Without the impact of these fortification strategies, the annual burden of IDA is estimated at 242,100 disability adjusted life years (DALYs) and 978.1 million USD. Wheat flour and condiment fortification contributed to a reduction of the IDA burden by approximately 5% each.

**Conclusion:**

In places with high prevalence of malaria and other infectious diseases, such as the Côte D’Ivoire, food fortification as a nutritional intervention should be accompanied with infectious disease prevention and control. The findings of this study provide additional input for policy makers about the magnitude of the impact and can support the conception of future fortification strategies.

## Introduction

In 2010, an estimated one-third of the world population was affected by anemia [1, 2], resulting in the loss of 68 million disability-adjusted life years (DALYs) during that year [1]. There has been progress in reducing the prevalence of anemia worldwide from 40.2 to 32.9% from 1990 to 2010 [1]. Southeast Asia recorded the greatest improvements, whereas sub-Saharan Africa achieved the lowest progress [1]. Despite the improvement, still over half of the burden of anemia arises from iron deficiency, accounting for over 35 million DALYs lost [2, 3]. This estimation is based on a widely used attribution of 50% of anemia to iron deficiency. The latest World Health Organization (WHO) report on nutritional anemia is based on this proportion [4]. This share has been challenged by a recent meta-analysis of 23 national surveys, which concluded that only about 25% of anemia among preschool children is attributable to iron deficiency, and 37% among women in reproductive age [5].

The burden associated with iron deficiency anemia (IDA) is the highest among nutritional deficiencies around the world surpassing all other nutritional deficiencies combined including protein-energy malnutrition [3]. In the Cote d’Ivoire, iron deficiency (41–63%) and IDA (20–39%) are highly prevalent among women and children [6].

Although income growth should induce improved nutritional status on the long-run, evidence from low-income countries showed a low-income elasticity of micronutrient demand, which means that income growth leads only to a marginal contribution to curb IDA prevalence, thus highlighting the need for additional strategies to accelerate the decline [7, 8]. Some governments implemented, in addition to educational, sanitary and health interventions to prevent and treat parasitic diseases, mandatory mass fortification of staple foods and iron supplementation programs [9–11]. In the Philippines, a study looking at the impact of mandatory fortification between 2003 and 2013 showed that the prevalence of anemia in Filipino children aged 1–4 years was reduced from 37.4 to 11.3% [12]. This was due to a combination of strategies that included food fortification and infection control. In Costa Rica, fortification of wheat flour and milk resulted in a marked reduction in anemia among women and children [13]. In particular, iron deficiency among children declined from 26.9 to 6.8% and IDA declined from 6.2% at baseline to undetectable levels.

In the Cote d’Ivoire, iron fortification of wheat flour became mandatory in 2007 as part of the fortification policy [10]. Despite its mandatory nature, subsequent surveys evaluating the impact of this policy showed that only around 50% of the flour samples complied with the mandated levels of iron fortification limiting the potential impact [11, 14]. Rohner et al. conclude that despite this policy, the prevalence of IDA remains high and that there is still a need for additional iron-fortified food vehicles to reduce the burden [11].

Next to staple and processed foods, condiments and seasonings are promising vehicles for iron fortification [15–17], because even vulnerable population groups consume these regularly. Data from a recent Fortification Rapid Assessment Tool (FRAT) survey across 12 countries in sub-Saharan Africa indicated that between 79 and 99% of respondents consumed bouillon cubes [18]. Some programs in Asia and Africa now use condiments and seasonings as vehicles to address micronutrient deficiencies [19, 20].

In the Cote d’Ivoire, a cross-sectional survey showed that 97% of women in child-bearing age consumed bouillon cubes [11]. The estimated mean consumption was 3.7 g/day among women of child-bearing age and 1.4 g/day among 6–23-month-old children. Although iron fortification of condiments in the Cote d’Ivoire is not mandatory, the voluntary iron fortification of the main commercial brand began in May 2013, adding 2.1 mg of iron per 3.3 g of bouillon, meaning that at least 90% of bouillons sold in the country has been fortified since. Hurrell et al. and Klassen et al. provide a thorough description of the mandatory and voluntary fortifications programs in Côte d’Ivoire and West and Central Africa [21, 22].

Burden of disease studies provides important scientific information to decision makers about the scale of a public health problem and its consequences. Analyzing to what extent different policies can help to alleviate the problem helps to compare alternatives. The objectives of this study are firstly to estimate the burden of Iron Deficiency Anemia as of 2014 in Cote D’Ivoire, and secondly to assess the contribution of iron-fortified flour and bouillon cubes to the reduction of the burden.

## Materials and methods

**Fig. 1:**
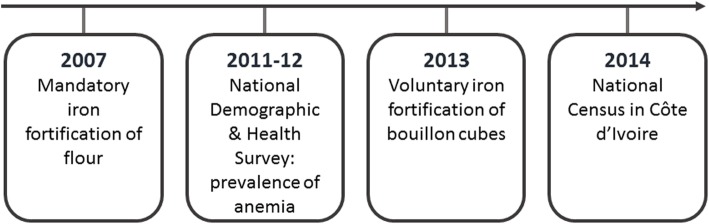
Sequence of fortification interventions and national surveys in Cote d’Ivoire

We estimate the burden of IDA as of 2014 considering the anemia prevalence (using the thresholds proposed by WHO), the share of anemia attributed to iron deficiency, efficacy of the fortification programs, and the population registered in the Ivorian Census in 2014. Because there are different dates between the initiation of the fortification programs and data collection, we consider three scenarios to estimate the burden of IDA. In the first scenario, we modeled a lower theoretical IDA prevalence than what was directly derived from the Demographic and Health Survey (Enquète Démographique et de Santé, DHS) 2011/12 because in 2014 the population had an increased iron intake from voluntary bouillon fortification program starting in May 2013. The second scenario reflects the prevalence directly derived from DHS 2011/12 data and takes into account the impact of the mandatory iron fortification of flour that initiated in 2007 but not the bouillon fortification. The third scenario, we model a higher IDA prevalence than the data derived from DHS 2011/12 deducing the iron intake from the wheat fortification program to estimate what would be the prevalence in the absence of both fortification programs. The sequence of the above points is summarized in Fig. 1.

The following paragraphs explain details of the model, the population subgroups used in the analysis, the prevalence estimation, and the considered impacts of fortification which allows us to estimate the contribution of flour and condiment iron fortification to the reduction of the health and economic burden.

### Model

To estimate the health and economic burden of IDA, we adopted the comparative risk assessment model (CRA). CRA models use aggregate-level and population-attributable fractions to illustrate how an intervention would influence the parameters describing the relationship between a risk factor and disease outcome would change following an intervention [23]. We consider iron fortification of bouillon and flour as an intervention to increase iron intake (reducing the risk factor for low dietary iron intake) thus lowering the iron deficiency anemia. As we later explain in “Prevalence of iron deficiency anemia” section, iron deficiency anemia has to be imputed from anemia prevalence using the percentage of anemia attributed to iron deficiency as in Côte D’Ivoire there is not a recent report at national level directly on IDA.

**Fig. 2:**
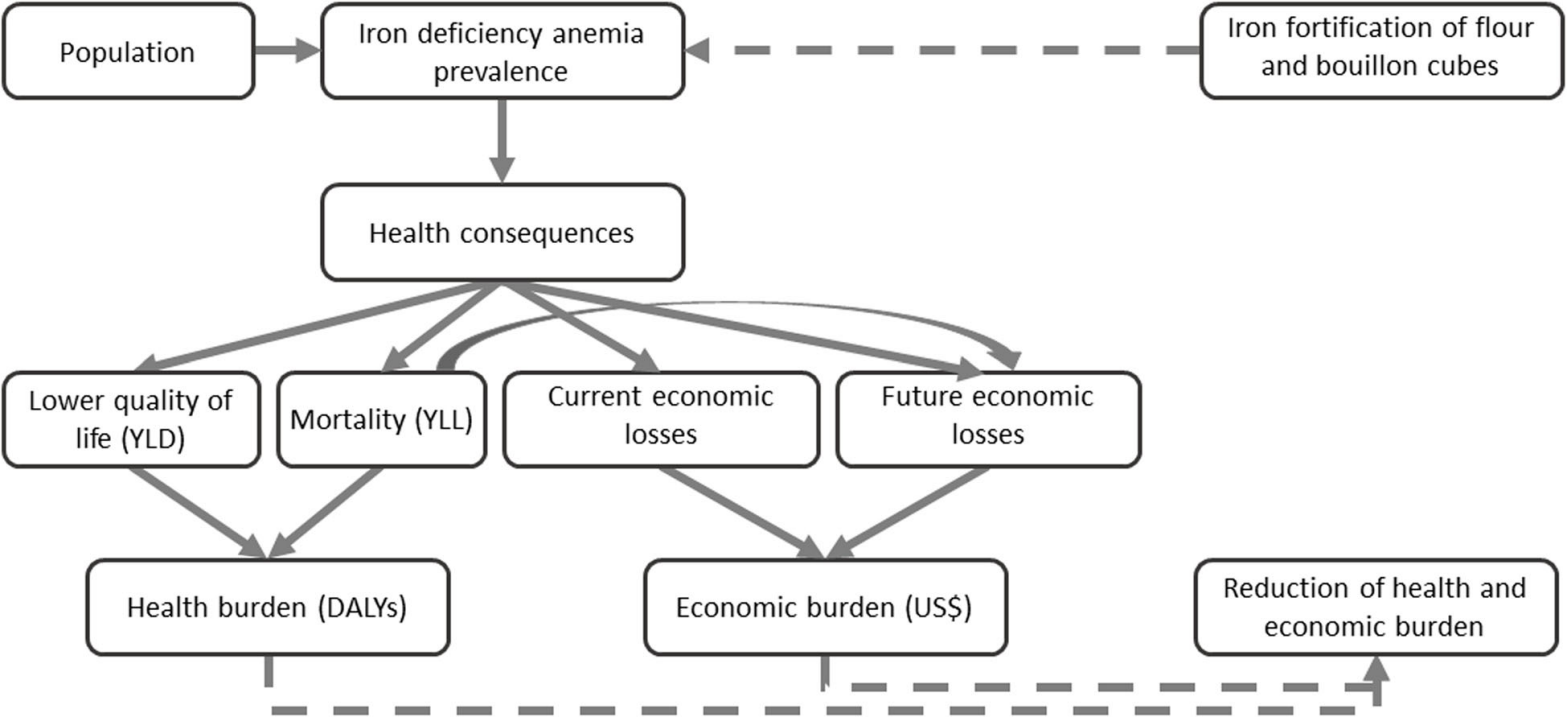
Estimating the economic and health burden of IDA

Our model is segmented in three age groups, children under 5 years old, school age children, and adults. For children under 5 years old, we followed an approach proposed by Wieser et al. [24]. For adults, we use similar methodology as Bagriansky et al. [25] and Alcazar [26]. For school age children, we built a model for the poorer learning and schooling outcomes linked to anemia [27–31] and its consequence on future productivity [32]. The model considers the prevalence of iron deficiency anemia for each specific population group, as well as its health consequences and estimates the economic and health burden of the disease (Fig. 2).

### Population

We considered in our model 21 million habitants between the age of 6 months and 64 years based on 2014 Ivorian Census [33]. Because both the prevalence and the health consequences of IDA differ by age, gender, and socioeconomic status, we split the population in our analysis into several groups. By age, we divide the population into three main groups: pre-school age children 6 months to 4 years old (2.7 million); school-age children 5 to 14 years old (6.2 million); and adults 15 to 64 years old (12.1 million). Within these age groups, we created additional subgroups. For pre-school age children, we modeled separately children 6 to 23 months and 24 to 59 months. For school age children, we considered each year cohort separately and distinguished between boys and girls. For adults, we created four sub-groups: men, non-pregnant women in reproductive age, pregnant women, and older women. Furthermore, we clustered each subgroup into ten socioeconomic strata (SES).

### Prevalence of iron deficiency anemia

We used raw data from the National Demographic and Health Survey (in French, Enquète Démographique et de Santé, DHS) 2011–2012 in the Cote d’Ivoire to estimate the prevalence of anemia in each population subgroup and SES. The socioeconomic stratification is based on a wealth score using weights on over 100 elements reflecting household assets and other dimensions of wealth through principal component analysis. This is the only survey in the Cote d’Ivoire which included population-wide information on hemoglobin concentrations from blood tests of pre-school age children (6 to 59 months) and adult women and men (15 to 49 years old). However, hemoglobin data were not available for school-aged children (5 to 14 year old) in the Enquète Démographique et de Santé (EDS) 2012. Therefore, we imputed the data based on results from a local study in Cote d’Ivoire that reported the prevalence of IDA in older versus younger children [6].

In our analysis, we were interested in modeling the prevalence of IDA by each SES cluster within each population subgroup. However, EDS was designed to estimate the prevalence of anemia at national level. Therefore, we used the regression analysis between hemoglobin and wealth index in each population subgroup to determine the mean hemoglobin concentration for each SES cluster. Then, using the mean, the standard deviation and assuming that hemoglobin concentrations are normally distributed, we were able to determine the prevalence and severity of anemia in each SES and population sub-group.

**Fig. 3:**
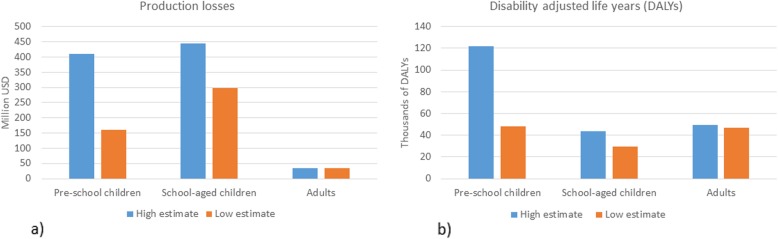
Comparison of the health and economic burden using high and low estimates on the attribution of anemia due to IDA (**a**) corresponds to the production losses among the two estimates while (**b**) corresponds to the DALYs

In our model, we adopted the figure proposed by Asorbayire et al. [6] and Kassebaum et al. [1] for the proportions of anemia due to iron deficiency which are the most referenced estimates in the literature. These were 64% for children under 5 years, 45% for school-age children, 39% for women, and 18% for adult men. We acknowledge that the attribution made by Asorbayire et al. [6] and Kassebaum et al. [1] has been recently discussed on publications of the BRINDA group [34–36] and Petry et al. [5] suggested lower estimates. In order to show implications of both estimates of the attribution of anemia due to iron deficiency, we compered the results using the low and higher estimates on Fig. 3 and in more details in the Appendix D.

### Health consequences of IDA

The three health consequences of IDA that we considered in the model are (a) cognitive impairment in infancy between 6 and 23 months, (b) child mortality between 6 to 59 months, and (c) reduced physical activity (weakness and fatigue) for all age groups [37–41]. Cognitive impairment in early childhood and child mortality are irreversible health consequences of IDA, while reduced physical activity can be reverted once hemoglobin levels are increased.

We did not include the consequences of maternal IDA on the increased risk of preterm labor, low infant birth weight, infantile anemia, and maternal mortality. We did so partly because data on these parameters are missing. Furthermore, it would have added considerable additional complexity to the model. Therefore, our estimation remains partial and conservative on costs as it probably underestimates certain aspects of IDA (Table 1).

**Table 1:** Prevalence of anemia and iron deficiency anemia for the age groups considered in the model

Age group	Anemia prevalence	Share due to ID^a^	IDA estimate	IDA highest SES	IDA lowest SES
Pre-school children	74.8%	64.0%	47.9%	37.8%	52.5%
School age children	56.1%	49.0%	27.5%	21.7%	30.1%
Women	54.9%	39.0%	21.4%	18.8%	22.3%
Men	29.7%	18.0%	5.3%	4.5%	6.0%

*IDA* iron deficiency anemia, *ID* iron deficiency, *SES* socioeconomic strata; pre-school children from 0.5 to 4 years old; school age children from 5 to 14 years old: adults men and women from 15 to 64 years old

^a^The prevalence of anemia was estimated from row data of the Demographic and Health Survey 2011–2012 in Côte D’Ivoire. The share of iron deficiency anemia is estimated based on Kassebaum, et al. and Asorbayire, et al. [1, 6]

### The health burden of IDA

To quantify the burden of the health consequences of IDA, we applied the methodology established by the Global Burden of Disease and used the disability weights of Stein et al. adjusted by Plessow et al. [2, 42–45]. Table 2 summarizes the key figures used in the calculus. For estimating the cognitive decline due to moderate and severe IDA among 6–23-month-old infants and the mortality of 6–59-month-old infants, we follow the approach of Wieser et al., consider estimates of Lozoff et al. (2006) on the reduction of intelligence quotient (IQ), and of Brabin et al. (2001) on child mortality [24, 37, 39].

**Table 2:** Parameters used for estimating the health burden (in DALYs) due to IDA by Plessow et al. [38]

Health consequence	Severity	Disability weights	Age-group	Source
Impaired physical activity	Mild	0.005	All	Solomon J. A et al., Plessow et al., and Murray et al. [2, 43, 45]
	Moderate	0.058	All	
	Severe	0.164	All	
Cognitive impairment	Moderate	0.0078	6–23 months (pre-school children)	Plessow et al. and Murray et al. [43, 45]
	Severe	0.031	6–23 months (pre-school children)	
All-cause mortality	Severe	1.0	6–59 months (pre-school children)	Plessow et al. and Murray et al. [43, 45]

In the model, we calculate the life-long burden of the irreversible health consequences of IDA (cognitive impairment and child mortality) beyond year 2014 (the modeled year). For child mortality, we consider the total years of life lost (remaining life expectancy at children’s age). For mental impairment, we took into account the estimated number of years lived with this condition. In the model for the reversible health consequence, namely reduced physical activity, only the current year was considered.

### The economic burden of IDA (production losses)

The economic burden is driven by production losses as we did not include direct medical cost. We did not include direct medical costs because only a small fraction of IDA is treated in the Ivorian setting and therefore the costs are negligible. In Table 3, we present the key parameters used to quantify the economic burden associated with the health consequences of IDA. Our economic model is inspired by Horton and Ross [55]. Similar to them, we only consider production losses in estimating the economic burden.

**Table 3:** Parameters to estimate the production losses (economic burden) linked with IDA

Parameter	Value	Source
Life expectancy	58 years	World Factbook [46]
Age to begin working life	15 years	World Bank [47]
Age of ending working life	64 years	World Bank [47]
Mean monthly wage	65,575 XOF	National Statistics Cote D’Ivoire [48]
Monetary discount rate	3%	Smith [49]
Expected income growth	3.5%	World Bank [47]
Work force participation (net)	63%	World Bank [47]
Income distribution	SES 1: 1.9% to SES 10: 34.8%	UNU-WIDER [50]
Unadjusted intergeneration income correlation	0.55	Black, Solon [51, 52]
Education adjusted intergeneration income correlation	0.35	Black [51]
Returns on education	10.9%	Psacharopoulos [32]
Impact of anemia on school performance	− 10%	Hutchinson et al., Soemantri et al., [29, 30]
Impact of anemia on school attendance	− 5.3%	Bobonis et al., Hutchinson et al., Bobonis et al., Walker et al. [27, 29–31]
Relative risk of school absenteeism on school drop-outs	1.8	Walker et al. [31]
Impact of 1 standard deviation reduction in cognitive score on future wages	− 8%	Psacharopoulos [53]
Impact of IDA on productivity of moderate manual labor	− 5%	Basta [51]
Impact of IDA on productivity of intense manual labor	− 17%	Basta [54]

*IDA* iron deficiency anemia, *SES* socioeconomic strata, *XOF* West African CFA Franc (CFA stands for Communauté Financière Africaine); Work force participation (net): excludes those unemployed

We quantify two types of production losses, those that are occurring in the year that we are modeling and those, which take place in the future. In the model, current production losses encompass lower wages of workers due to inferior productivity because of weakness and fatigue associated with anemia. For estimating future production losses associated with IDA, the model considers lower human capital accumulation, originated from mortality among 0.5–4-year-old children, cognitive impairment of 6–23-month-old pre-school children, and lower retention of knowledge and school drop-out of 5–14-year-old children. To compute the future production losses of pre-school children, we followed the approach developed by Wieser et al. [24] and Plessow et al. [43]. Most of the costs incurred by this age group arise from cognitive impairment due to moderate and severe anemia reflected in reduced future wages. To estimate the losses due to cognitive impairment, we first estimate the IQ loss for infants suffering severe and moderate anemia [36]. Then based on the association between IQ and wages, we calculate the expected average wage difference associated with lower IQ, multiply by the expected years that a child would work, and discount to obtain the net present value. To predict baseline future wages, Wieser et al. used the average wage for the country alongside an expected growth trajectory [24].

To estimate the economic burden of IDA for children aged 5–14 years, we count with the negative effect of anemia on school learning and consider 10% lower retention of knowledge, higher school absenteeism, and early school dropouts leading to reduced life-long productivity (Table 3) [30, 31]. For this, we applied returns on education figures originating from sub-Saharan Africa (Table 3) [32].

In estimating the productivity loss for adults, we calculated lower productivity of manual labor using figures of Basta et al. [54] (Table 3), similarly to Horton et al. [55]. Additionally, we took into account the prevalence of IDA by SES, age, gender, and for women by pregnancy status.

In order to obtain a more precise estimation of production losses, we took into account income inequality by SES, as well as future wage inequalities using estimates of intergenerational income correlation [2, 50]. The calculation of production losses linked to lower school performance is detailed in Appendix A.

### Calculating the reduction of the health and economic burden linked to iron fortification

We considered the following aspects to estimate the impact of fortification. First, we calculated the additional milligrams of iron intake by age-groups because of the fortification intervention. We based our estimation on consumption data by age-group and fortification levels of the food vehicle. Second, we translated the additional milligrams of iron into an estimated increase in hemoglobin based on published effectiveness of the iron compound used, and adjusted it for other bioavailability aspects, such as the rest of the diet. Third, we estimated the hypothetical prevalence of IDA based on population level hemoglobin distribution taking into account the fortification effect. Finally, we estimated the burden for the hypothetical IDA prevalence.

The input parameters for the model are summarized in Table 4. The consumption of fortified wheat flour and the levels of iron fortification was calculated based on a survey from Cote d’Ivoire [11]. For condiments, an annual consumption of 6 billion servings (unpublished data) and 2.1 mg iron per serving is considered.

**Table 4:** Parameters used to estimate the effect of iron fortified flour and bouillon cubes on IDA

Parameter	Age-group	Value	Source
Additional iron intake			
Wheat flour	6–23 months Adult women	0.74 mg/day 2.63 mg/day	Own calculation based on Rohner et al. [11]
Bouillon cubes	6–23 months Adult women	0.89 mg/day 2.35 mg/day	
Increase in hemoglobin			
Wheat flour	6–23 months 24–59 months 5–64 years	0.561 g/L 0.798 g/L 1.034 g/L	Own calculation based on: Hess et al., Winichangoon et al. [56, 57]
Bouillon cubes	6–23 months 24–59 months 5–64 years	0.673 g/L 0.799 g/L 0.924 g/L	

The impact of iron-fortification on increasing hemoglobin levels was calculated based on the meta-analysis by Hess et al. [56]. This meta-analysis reported an enhanced hemoglobin concentration of 7.4 g/L in the study population (aged 5–50 years) by adding on average 10.6 ± 4.7 mg of iron to the diet per day.

Considering the fortification dose per serving of bouillon cubes and the average number of servings consumed per day per person (1.1), the effect over several months could result in a hemoglobin increase of 1.6 g/L. However, because there is a difference in the bioavailability (percentage of the iron absorbed by the body) of different iron compounds used in the clinical trials, we selected a sub-sample of publications [57–59] from the Hess review that used iron pyrophosphate or similar compound suitable for the fortification of bouillon cubes. As a consequence, the impact of a condiment (i.e., bouillon cubes) fortified at 15% of the Codex nutrient reference values (NRVs) (i.e., 15% of 14 mg = 2.1 mg iron per serving) consumed at 1.1 servings per day over a long period of time could result in a hemoglobin increase of 0.924 g/L in adults and school-aged children. For children, 6–23 months, the reported consumption of bouillon is 1.4 g/day [11] which represent 0.891 additional mg of iron a day equivalent to 12.4% NRV. This could lead to an increase of the hemoglobin concentration of 0.673 g/L. In the absence of information on what fortifying agent is used exactly in wheat flour, we assumed similar bioavailability as for the condiments. The approach detailed above for condiments was applied also to wheat flour adjusting for level of consumption and fortification.

## Results

In Tables 5 and 6, we present the results from the three scenarios described in the previous: (1) current situation, after condiment and flour fortification; (2) after flour and before condiment fortification; and (3) before flour fortification. Table 5 presents the estimated economic burden of IDA and Table 6 presents for the same scenarios for both the discounted and the undiscounted health burden. The results presented in Tables 5 and 6 build on figures of Table 1, using estimates of Kassebaum et al. and Asorbayire et al. [1, 6] for the attribution of anemia due to ID. Additionally, we present figures for the attribution of anemia due to ID as published by Petry et al. in Appendix D (Tables 9 and 10) [5].

**Table 5:** Economic burden: production losses before and after iron fortification of wheat flour and condiments by age-group

	Pre-school children	School-aged children	Adults	Total
Million USD				
After condiment and flour fortification (mean (95% CI))	411 [255.5: 623.1]	444.3 [221: 750.6]	34.6 [26.1: 44.2]	889.9 [581.5: 1306.8]
After flour and before condiment fortification (mean (95% CI))	425.4 [264.8: 644.5]	471.5 [234.6: 796.3]	36.3 [27.5: 46.4]	933.1 [609.2: 1371]
Before flour fortification (mean (95% CI))	437.6 [272.6: 662.6]	502.4 [250.1: 847]	38.2 [28.9: 48.8]	978.1 [636.8: 1438.1]
Absolute change attributable to flour fortification (mean)	12.2	30.9	1.9	45
Absolute change attributable to condiment fortification (mean)	14.4	27.2	1.7	43.2
% change				
Reduction attributable to flour fortification	2.8%	6.1%	5.0%	4.6%
Reduction attributable to condiment fortification	3.4%	5.8%	4.7%	4.6%

*CI* confidence interval. This refers to constructed 95% confidence intervals as calculated in the probabilistic sensitivity analysis. Pre-school children: 0.5 to 4 years; school age children: 5 to 14 years; adult men and women: 15 to 64 years

**Table 6:** Health burden: disability adjusted life years (DALYs) before and after iron fortification of wheat flour and condiments by age-group

	Pre-school children	School-aged children	Adults	Total
Discounted health burden				
‘000 DALYs				
After condiment and flour fortification (mean (95% CI))	121.9 [92.4: 157.8]	43.8 [25.3: 66.5]	49.1 [29.2: 74]	214.7 [156.7: 286.2]
After flour and before condiment fortification (mean (95% CI))	128.3 [97.5: 165.8]	47.3 [27.4: 71.7]	52.6 [31.4: 79.2]	228.1 [166.7: 304]
Before flour fortification (mean [95%CI])	134.2 [102.4: 173.2]	51.3 [29.9: 77.8]	56.6 [34: 85.1)	242.1 [177.1: 322.8]
Absolute change attributable to flour fortification (mean)	5.9	4.1	4.1	14
Absolute change attributable to condiment fortification (mean)	6.4	3.5	3.5	13.4
% change				
Reduction attributable to flour fortification	4.4%	7.9%	7.2%	5.8%
Reduction attributable to condiment fortification	4.9%	7.3%	6.7%	5.9%
Undiscounted health burden				
‘000 DALYs				
After condiment and flour fortification (mean (95% CI))	205.4 [153.3: 267.4]	43.8 [25.3: 66.5]	49.1 [29.2: 74]	298.2 [224: 384.7]
After flour and before condiment fortification [mean (95% CI))	216.4 [161.9: 281.6]	47.3 [27.4: 71.7]	52.6 [31.4: 79.2]	316.3 [237.9: 407.1]
Before flour fortification (mean (95% CI))	226.5 [169.7: 294.1]	51.3 [29.9: 77.8]	56.6 [34: 85.1]	334.4 [251.7: 430.7]
Absolute change attributable to flour fortification (mean)	10	4.1	4.1	18.2
Absolute change attributable to condiment fortification (mean)	11.1	3.5	3.5	18
% change				
Reduction attributable to flour fortification	4.4%	7.9%	7.2%	5.4%
Reduction attributable to condiment fortification	5.1%	7.3%	6.7%	5.7%

*DALYs* disability adjusted life years, *CI* confidence interval. This refers to constructed 95% confidence intervals as calculated in the probabilistic sensitivity analysis. Pre-school children: 0.5 to 4 years; school age children: 5 to 14 years; adult men and women: 15 to 64 years

We estimate the annual economic and health burden of IDA in 2014 in the Ivorian population (from 6 months to 64 year-old) to be in the range of 582 and 1307 million USD and between 156,700 and 286,200 DALYs (Tables 5 and 6). These values include the impact of the additional iron intake from fortifying wheat flour and bouillon cubes. The total average annual cost of IDA, 890 million USD, represents 2.5% of the Ivorian Gross Domestic Product and a health burden of 214,700 DALYs is equivalent to 5141 full life spans in good health lost each year. Pre-school age children (6–59 months) represented 15% of the population in our model, carrying 46% of the economic losses and 57% of the discounted health burden linked to IDA. School age children (5 to 14 years old) represented 29% of the population carrying 50% of the economic losses linked to the impact of IDA on schooling and 20% of the discounted health burden. Overall, adults (men and women 15 to 64 years old) represent 56% of the population, but only 4% of the economic losses and 23% of the discounted health burden.

Overall, mandatory flour fortification as surveyed in 2010 translates into 45 million USD in economic and 14,000 discounted DALY gains in reducing the IDA burden annually (Tables 5 and 6). The introduction of iron-fortified condiments in 2013, based on surveyed consumption and sales volume figures, contributed to a reduction of the annual burden with 43.2 million USD economic and 13,400 DALYs discounted health gains. It appears that the contribution of mandatory wheat flour fortification to the reduction of the IDA burden is in a similar range, with 45 million USD and 14,000 DALYs.

These results are highly dependent on the share of anemia attributed to ID. Using the attribution of anemia of 25% for pre-school age children and 30% for school age children, 37% for women and 18% for men by Petry et al. [5] means that the annual IDA burden is estimated at 491 million USD and 124 thousand DALYs, nearly half of the previous estimate.

### Probabilistic sensitivity analysis

We use Monte Carlo simulation to run a multivariate probabilistic sensitivity analysis (PSA) to generate synthetic confidence interval for our estimations. The underlying distributions and confidence intervals of the parameters for the probabilistic sensitivity analysis are summarized in the Appendix B and C.

**Fig. 4:**
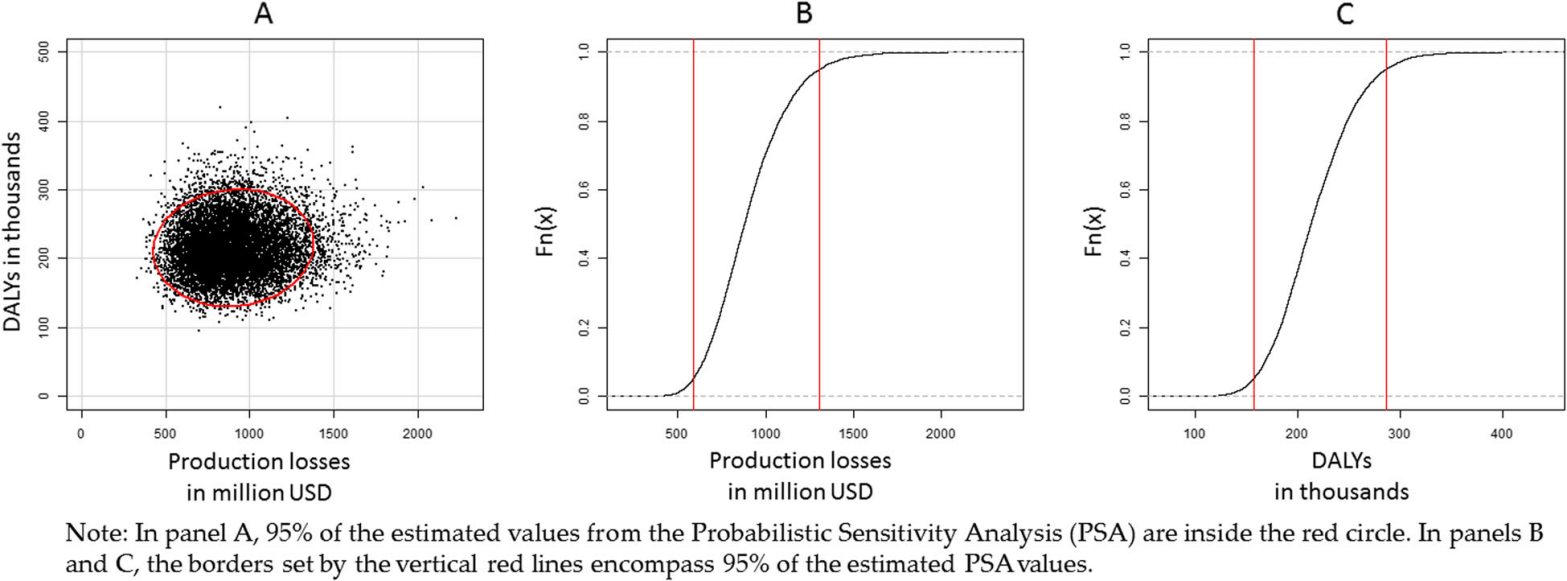
Probabilistic sensitivity analysis

Figure 3 plots the results of 10,000 model runs for the total discounted health and economic burden for 2014, the latter expressed in production losses. The resulting oval area in panel A circled with red is the set of 95% chance of falling within the limits. The oval shape indicates that there is more variability in the estimation of the economic burden than in the health burden, which can be explained by the extensive number of influencing variables of the economic part of the model. Panel B shows that the production losses vary in 95% of the cases from 581 million USD to 1.3 billion USD. Panel C illustrates that in 95% of the cases, the health burden is between 157 and 286 thousand DALYs. The larger synthetic confidence intervals in the production losses result from higher variance on scenarios that predict the economic and wage growth in Cote D’Ivoire. Identical probabilistic sensitivity analyses were ran in all scenarios, details of which are not presented here (Fig. 4).

## Discussion

Iron fortification of wheat flour and condiments are strategies to reduce the burden of IDA [19, 56, 60]. Our objective was to provide an estimate on the extent to which these can contribute towards reducing the burden of IDA in Cote D’Ivoire. We modeled the health and economic effects of food fortification for the population from 6 months to 64 years of age by combining different published sources. To our knowledge, this is the first study reporting the contribution of fortified wheat flour and condiment consumption to reducing the burden of IDA in Cote d’Ivoire. We expressed the burden in terms of economic losses (productivity losses in USD) and health impact (number of DALYs).

Horton and Ross’s estimation of the burden of iron deficiency in a sample of ten developing countries was 4% of the GDP, which is almost double our estimate [55]. This may arise from our more conservative assumption on the prevalence of iron deficiency anemia. We also excluded the burden of iron deficiency without anemia in the absence of accurate national level figures. Taking into account these differences, the magnitude of our estimations is comparable to the above study.

Our model takes into consideration socioeconomic groups for the prevalence of IDA and for the estimation of SES specific wages. This approach is different from previous estimations on the burden of IDA, as they used only mean wages [25]. However, the intake of iron from fortified food vehicles examined in this study is included with a mean value. The level of iron fortification of condiments is standard and survey data on their intake suggests that their daily consumption is relatively stable across different socioeconomic groups. For wheat flour, we have to consider that only 50% of the samples in rural areas were adequately fortified [11]. Furthermore, rural areas are inhabited largely by the poorest 40% of the households (SES 1–4) and lower socioeconomic strata carry a higher burden of IDA. This in practice means that flour fortification of iron benefits more the urban, hence wealthier households. Using SES-specific wages results in a cost burden that is 12% lower than using a mean wage (since higher SES groups have higher income but lower IDA prevalence). However, because of the uneven iron intake from wheat flour through SES groups, production losses are likely to be underestimated for this food vehicle in our model. Considering the above points and that we used similar fortification level of wheat flour for both urban and rural population groups, our calculation most likely overestimates the impact of wheat flour fortification on reducing the health burden.

There are several limitations to our study. Little data is available on the prevalence of IDA in the Côte d’Ivoire; therefore, we generated a model based on data from the EDS 2012, which only reports anemia levels based on hemoglobin and not iron deficiency anemia. We used figures of Asorbayire and Kassebaum to estimate the share of anemia associated with iron deficiency [1, 6]. However, Petry et al. in a recent systematic review suggest that the Kassebaum figures could be too high leading to an overestimation of the burden [4]. Therefore, we have run the model with the figures proposed by Petry et al. [5], which would lead to an annual IDA burden estimate of 491 million USD and 124 thousand DALYs (presented in details in Appendix D), a much smaller figure than using the Kassebaum et al. and Asorbayire et al. estimates.

We did not include adverse effects of additional iron intake linked to higher risk of malaria and bacterial infection as the level of fortification remains low balancing the effectiveness and safety objectives [60]. The adverse effect observed from additional supply of iron are higher dosage related to supplementation rather than low dose fortification [60]. Additional studies had highlighted the need of low dose fortification with high bioavailable iron to minimize the safety concerns and reducing iron deficiency in low-income countries [61].

A further limitation of our analysis is that the DHS did not include any information on hemoglobin concentration among school-aged children. Therefore, we had to extrapolate the IDA estimates for this sub-population based on an Ivorian study that compared the prevalence of IDA in older versus younger children [6]. There is the possibility that the data used for our estimates were over- or under-reported. Many factors can influence the absorption of iron, such as the deficiency of other micronutrients, the diet, or infections [62–65], which may affect our estimates. These uncertainties are reflected to some extent in the PSA; nevertheless, considerably more certain estimates could be generated if there were nationwide representative surveys not just registering hemoglobin, but also other biomarkers, which would enable a more accurate estimation of the prevalence of IDA. Our model estimates the production losses linked with education using figures of Psacharoupulos [32]. However, other authors suggest that his estimates are high and propose lower values [66]. It means that our model may lead to an overestimation of the production losses among school-age children. Finally, our study considered the average additional iron intake for each food vehicle on the target population. Using mean values for iron intake is a combination of the level of consumption and the fortification level. This does not allow us to evaluate the appropriateness of the food vehicle based on the coverage of its consumption. For instance, a large portion of the wheat flour consumed in Côte D’Ivoire is in a non-fortifiable form (not processed) compared with bouillons that are fortified. As Aaron et al. emphasize, the success of fortification programs is linked with the convenience of the food vehicle, the regularity of intake and of consumption levels, which need to be assessed locally as they vary by country and by food vehicle [67].

Our results support the use of fortified condiments as a potential strategy for addressing IDA in the Côte d’Ivoire in addition to wheat flour iron fortification. Iron fortification of condiments such as bouillon cubes presents a valuable option for improving micronutrient intake at the population level across different socioeconomic groups. Policy makers and nutrition program managers should encourage the use of fortified over non-fortified foods and condiments and ensure that the food industry takes action with respect to the fortification of widely consumed staple foods and condiments [68]. Additionally, because of the uncertainty around the proportion of anemia attributable to iron deficiency, it is important to develop comprehensive approaches to tackle the anemia and IDA burden, where nutrition interventions are complemented with improved sanitation, malaria, and other infectious disease prevention and control measures, as in combination they may generate greater benefits than each program individually. The World Health Organization recommends a multi-faceted approach, including fortification, increasing dietary diversity, and supplementation for groups at risk (such as pregnant women) [41]. Nevertheless, the use of widely consumed fortified food vehicles presents a feasible additional food-based mechanism for augmenting iron status in developing countries such as the Côte d’Ivoire.

## Conclusions

IDA remains a major health problem in the Côte d’Ivoire, resulting in a high health and economic burden representing 2.5% of the Ivorian gross domestic product and a health burden equivalent to 5141 full life spans in good health lost each year. Despite current fortification strategies, there is still an unmet need for effective interventions that can improve iron status at the population level. In this analysis, we present estimates linked to the iron fortification of wheat flour, which was introduced as a mandatory measure in Cote d’Ivoire, and to condiments, which is a widely consumed food vehicle. Previous studies have shown that although the fortification of wheat flour is mandatory, fortification levels are lagging behind the mandatory levels and consumption levels in a fortifiable form are low. If there was a better vehicle for mandatory fortification, the impact could be improved. Our results indicate that iron fortification of condiments (namely, bouillon cubes) presents a feasible additional strategy for improving iron status complementing mandatory fortification [69].

## Data Availability

The study is primary based on the raw data of the Demographic Health Survey 2012 in Côte D’Ivoire. Data are publically available on the DHS website https://www.dhsprogram.com.

## Appendix A

### Calculation of production losses related to lower school performance

The calculations of production losses related to lower school performance are modeled by SES and by year cohort.

Calculation of production losses for children attending school with IDA who do not drop out of school: First, we count the number of children at given age attending school in that year and multiply this with the prevalence of IDA of children at any given age. This determines the number of children with IDA at every year. Then this figure is multiplied by 0.1, which is the estimated school loss for every student with IDA because of lower knowledge retention and higher absenteeism. The result is then multiplied by the average lifelong value of a year of education for a child at any given age. This is a function of several parameters including returns on education, expected future wages and working life span adjusted with a discount rate.

Calculation of production losses for children attending school with IDA who drop out of school: The total number of dropouts linked to IDA every school year is estimated using the population attributable fraction. Then we calculate the years of school lost due to early school leaving. The result is multiplied by the average lifelong value of a year of education for a child at any given age, which is a function of various variables as listed before.

## Appendix B

**Table 7:** Underlying distributions and confidence intervals of the parameters for the probabilistic sensitivity analysis

Parameters	Baseline values	95% CI	Distribution	References
Share of anemia due to IDA				
Pre-school-children %	64	(0.57: 0.70)	Beta	Kassebaum et al. and Asorbayire et al. [1, 6]
School age children %	45	(0.39: 0.51)	Beta	
Women %	39	(0.34: 0.44)	Beta	
Men %	18	(0.15: 0.20)	Beta	
Impact of anemia in preschool children				
Relative risk of mortality	2.19	(1.68: 3.36)	Lognormal	Brabin et al. [37]
IQ loss due to IDA (points)	9	(5.1: 13.4)	Gamma	Lozoff et al. [39]
Impact of IQ losses on wages %	8	(0.05: 0.01)	Beta	Psacharopoulos [53]
Intergeneration income correlation^a^	0.55	(0.50: 0.59)	Beta	Black, Solon [51, 52]
Impact of anemia in school children				
Relative risk to dropout (risk)	1.25	(1.01: 1.45)	Lognormal	Soemantri et al., Hutchinson et al., Bobonis et al., and Walker et al. [27, 30–33]
Reduced learning at school %	10	(0.087: 0.116)	Beta	Soemantri et al. and Hutchinson et al. [29, 30]
Intergeneration income correlation^a^	0.35	(0.31: 0.38)	Beta	Black [51]
Returns on education %	10.9	(0.079: 0.139)	Lognormal^b^	
Impact of anemia in adults on wages				
Moderate manual labor %	5	(0.045: 0.057)	Lognormal^b^	Basta [54]
Additional loss intense manual labor %	12	(0.11 0: 0.136)	Lognormal^b^	
Disability weights (DW) in score units				
Anemia mild (DW)	0.005	(0.002: 0.023)	Beta	Plessow et al. and Murray et al. [42, 43, 45]
Anemia moderate (DW)	0.058	(0.038: 0.086)	Beta	
Anemia severe (DW)	0.164	(0.112: 0.228)	Beta	
Intellectual disability mild (DW)	0.031	(0.018: 0.049)	Beta	
Intellectual disability moderate (DW)	0.08	(0.053: 0.114)	Beta	
Other parameters				
Life expectancy (years)	58	(56.2: 60.8)	Gamma	World Factbook [46]
Working life begins (years)	15	(13.2: 18.1)	Gamma	World Bank [47]
Working life ends (years)	65	(60.6: 71.4)	Gamma	World Bank [47]
Labor force participation %	55	(0.497: 0.571)	Beta	World Bank [47]
Projected income growth %	3.5	(0.039: 0.031)	Normal	World Bank [47]
Interest rate %	3	(0.034: 0.026)	Normal	Smith [49]
Mean monthly wage (CFA | 2015)	64,575	(54,575: 74,575)	Gamma	National Statistics Cote D’Ivoire [48]

^a^Intergenerational income correlation coefficient is unadjusted for years of education on pre-school children where for school age children is adjusted for education

^b^The percentages of those variables are converted into logarithms Log(var + 1) for PSA analysis

## Appendix C

**Table 8:** Table of the parameters used in the impact of fortified wheat flour and bouillon in hemoglobin in Côte D’Ivoire

Parameter description	Value	95% CI	Reference
Consumption per capita			
Bouillon cubes WRA g/day	3.7	(3.5: 3.9)	Rohner et al. [11]
Bouillon cubes 6–23 m g/day	1.4	(1.2: 1.5)	
Wheat flour a WRA g/day	125	(112: 138)	
Wheat flour a 6–23 mg/day	33	(29.2: 36.8)	
RNI and mg of iron per capita in WRA			
Iron as % of RNI from wheat flour	17.8	(15.7: 19.8)	Rohner et al. [11]
Iron as % of RNI from bouillon	15.9	(14.2: 17:4)	
Mg/day of iron from wheat flour	2.634	(2.32: 2.93)	
Mg/day of iron from bouillon cube	2.355	(2.01: 2.73)	
RNI and mg of iron from fortified vehicle 6–23 m			
Iron as % of RNI from wheat flour	10.6	(12.2: 9.1)	Rohner et al. [11] and WHO/FAO [70]
Iron as % of RNI from bouillon	12.4	(10.9: 13.9)	
Mg/day of iron from wheat flour	0.743	(0.54: 0.95)	
Mg/day of iron from bouillon cube	0.891	(0.65: 1.23)	
Recommended nutrient intakes (RNIs)			
RNI iron WRA in mg/day	14.8		WHO/FAO [70]
RNI iron 6–23 M in mg/day	7.2		
Increase in hemoglobin due to additional iron intake			
Δ Hb g/L in WRA from wheat flour	1.034	(0.84: 1.24)	Own calculation based
Δ Hb g/L in 6–23 m from wheat flour	0.561	(0.45: 0.67)	Hess et al.,
Δ Hb g/L in 24–59 m from wheat flour	0.798	(0.65: 0.96)	Winichangoon et al. [56, 57]
Δ Hb g/L in WRA from bouillon	0.924	(0.75: 1.11)	
Δ Hb g/L in 6–23 m from bouillon	0.673	(0.55: 0.81)	
Δ Hb g/L in 24–59 m from bouillon	0.799	(0.65: 0.96)	

*CI* confidence interval, WRA women in reproductive age, *RNI* recommended nutrient intakes, *Hb* hemoglobin

## Appendix D

### Tables using share of anemia attributed to 508 iron deficiency by Petry et al.

**Table 9:** Economic burden: production losses before and after iron fortification of wheat flour and condiments by age-group using anemia attributed to iron deficiency by Petry et al.

	Pre-school children	School-aged children	Adults	Total
million USD				
After condiment and flour fortification (mean (95% CI))	160.8 [101.5: 240.8]	297.3 [145.1: 511.9]	33.4 [25.3: 42.6]	491.5 [312.5: 737.6]
After flour and before condiment fortification (mean (95% CI))	166.4 [105.3: 249.1]	315.6 [154.1: 542.9]	35.1 [26.6: 44.7]	517 [328.2: 776.4]
Before flour fortification (mean (95%CI))	171.2 [108.4: 256.2]	336.3 [164.4: 578.1]	36.9 [28: 47]	544.5 [344.5: 818.3]
Absolute change attributable to flour fortification (mean)	4.8	20.7	1.8	27.5
Absolute change attributable to condiment fortification (mean)	5.6	18.3	1.7	25.5
% change				
Reduction attributable to flour fortification	2.80%	6.16%	4.88%	5.05%
Reduction attributable to condiment fortification	3.48%	6.16%	5.09%	5.19%

*CI* confidence interval. This refers to constructed 95% confidence intervals as calculated in the probabilistic sensitivity analysis. Pre-school children: 0.5 to 4 years; school age children: 5 to 14 years: adult men and women: 15 to 64 years

Using the attribution of anemia to iron deficiency of 25% for pre-school age children and 30% for school age children, 37% for women and 18% for men by Petry et al. [5]

**Table 10:** Health burden: disability adjusted life years (DALYs) before and after iron fortification of wheat flour and condiments by age-group using lower share of anemia attributed to iron deficiency

	Pre-school children	School-aged children	Adults	Total
Discounted health burden				
‘000 DALYs				
After condiment and flour fortification (mean (95% CI))	47.8 [36.9: 60.5]	29.2 [16.3: 45.7]	46.8 [27.9: 70.4]	123.8 [86: 170.3]
After flour and before condiment fortification (mean (95% CI))	50.3 [39: 63.6]	31.5 [17.7: 49.3]	50.1 [30: 75.3]	131.9 [91.8: 181.5]
Before flour fortification (mean (95% CI))	52.6 [40.9: 66.5]	34.2 [19.2: 53.4]	54 [32.4: 81.1]	140.9 [98: 193.8]
Absolute change attributable to flour fortification (mean)	2.3	2.7	3.9	9
Absolute change attributable to condiment fortification (mean)	2.5	2.3	3.3	8.1
% change				
Reduction attributable to flour fortification	4.37%	7.89%	7.22%	6.39%
Reduction attributable to condiment fortification	4.97%	7.30%	6.59%	6.14%

*DALYs* disability adjusted life years, *CI* confidence interval. This refers to constructed 95% confidence intervals as calculated in the probabilistic sensitivity analysis. Pre-school children from 0.5 to 4 years old; school age children from 5 to 14 years old; adults men and women from 15 to 64 years old

Using the attribution of anemia to iron deficiency of 25% for pre-school age children and 30% for school age children, 37% for women and 18% for men by Petry et al. [5]

## Abbreviations

CAF: Franc d’Afrique Centrale
Codex: Alimentarius International Food Code
CRA: Comparative risk assessment
DALY: Disability adjusted life years
DW: Disability weight
EDS: Enquète Démographique et de Santé
FAO: Food and Agriculture Organization
FRAT: Fortification Rapid Assessment Tool
GAIN: Global Alliance for Improved Nutrition
Hb: Hemoglobin
IDA: Iron deficiency anemia
IQ: Intelligence quotient
NRV: Nutrient reference values
PAF: Population attributable fractions
PIPAF: Programme Ivoirien de la Promotion des Aliments Fortifiés
PSA: Probabilistic sensitivity analysis
RDA: Recommended daily allowance
RNI: Recommended nutrient intakes
RR: Relative risk
SD: Standard deviation
SES: Socioeconomic strata
UNI-WIDER: United Nations University - World Institute for Development Economics Research
USD: United States Dollar
WHO: World Health Organization
WRA: Women in reproductive age
